# Stillbirth 2010–2018: a prospective, population-based, multi-country study from the Global Network

**DOI:** 10.1186/s12978-020-00991-y

**Published:** 2020-11-30

**Authors:** Elizabeth M. McClure, Sarah Saleem, Shivaprasad S. Goudar, Ana Garces, Ryan Whitworth, Fabian Esamai, Archana B. Patel, Shiyam Sunder Tikmani, Musaku Mwenechanya, Elwyn Chomba, Adrien Lokangaka, Carl L. Bose, Sherri Bucher, Edward A. Liechty, Nancy F. Krebs, S. Yogesh Kumar, Richard J. Derman, Patricia L. Hibberd, Waldemar A. Carlo, Janet L. Moore, Tracy L. Nolen, Marion Koso-Thomas, Robert L. Goldenberg

**Affiliations:** 1 Social, Statistical and Environmental Health Sciences, RTI International, 3040 Cornwallis Rd, Durham, NC 27709 USA; 2 Aga Khan University, Karachi, Pakistan; 3 KLE Academy Higher Education and Research J N Medical College Belagavi, Belagavi, Karnataka India; 4 Instituto de Nutrición de Centroamérica y Panamá, Guatemala City, Guatemala; 5 Moi University School of Medicine, Eldoret, Kenya; 6 Lata Medical Research Foundation, Nagpur, India; 7 University Teaching Hospital, Lusaka, Zambia; 8 Kinshasa School of Public Health, Kinshasa, Democratic Republic of Congo; 9 University of North Carolina at Chapel Hill, Chapel Hill, NC USA; 10 Indiana School of Medicine, University of Indiana, Indianapolis, IN USA; 11 University of Colorado School of Medicine, Denver, CO USA; 12 Thomas Jefferson University, Philadelphia, USA; 13 Boston University School of Public Health, Boston, MA USA; 14 University of Alabama at Birmingham, Birmingham, AL USA; 15 Eunice Kennedy Shriver National Institute of Child Health and Human Development, Bethesda, MD USA; 16 Department of Obstetrics and Gynecology, Columbia University School of Medicine, New York, NY USA

**Keywords:** Stillbirth, Low-middle income countries, Obstetric care, Global Network

## Abstract

**Background:**

Stillbirth rates are high and represent a substantial proportion of the under-5 mortality in low and middle-income countries (LMIC). In LMIC, where nearly 98% of stillbirths worldwide occur, few population-based studies have documented cause of stillbirths or the trends in rate of stillbirth over time.

**Methods:**

We undertook a prospective, population-based multi-country research study of all pregnant women in defined geographic areas across 7 sites in low-resource settings (Kenya, Zambia, Democratic Republic of Congo, India, Pakistan, and Guatemala). Staff collected demographic and health care characteristics with outcomes obtained at delivery. Cause of stillbirth was assigned by algorithm.

**Results:**

From 2010 through 2018, 573,148 women were enrolled with delivery data obtained. Of the 552,547 births that reached 500 g or 20 weeks gestation, 15,604 were stillbirths; a rate of 28.2 stillbirths per 1000 births. The stillbirth rates were 19.3 in the Guatemala site, 23.8 in the African sites, and 33.3 in the Asian sites. Specifically, stillbirth rates were highest in the Pakistan site, which also documented a substantial decrease in stillbirth rates over the study period, from 56.0 per 1000 (95% CI 51.0, 61.0) in 2010 to 44.4 per 1000 (95% CI 39.1, 49.7) in 2018. The Nagpur, India site also documented a substantial decrease in stillbirths from 32.5 (95% CI 29.0, 36.1) to 16.9 (95% CI 13.9, 19.9) per 1000 in 2018; however, other sites had only small declines in stillbirth over the same period. Women who were less educated and older as well as those with less access to antenatal care and with vaginal assisted delivery were at increased risk of stillbirth. The major fetal causes of stillbirth were birth asphyxia (44.0% of stillbirths) and infectious causes (22.2%). The maternal conditions that were observed among those with stillbirth were obstructed or prolonged labor, antepartum hemorrhage and maternal infections.

**Conclusions:**

Over the study period, stillbirth rates have remained relatively high across all sites. With the exceptions of the Pakistan and Nagpur sites, Global Network sites did not observe substantial changes in their stillbirth rates. Women who were less educated and had less access to antenatal and obstetric care remained at the highest burden of stillbirth.

**Study registration:**

Clinicaltrials.gov (ID# NCT01073475).

## Background

Globally, an estimated 2.1 million third trimester stillbirths (95% CI 1.8, 2.5) occurred in 2015, representing a decrease of nearly 50% since 1990 [1]. While this reduction is considerable, the overall rate of decrease in stillbirth lags well behind the rate of reductions that have occurred in under-5 mortality. Furthermore, nearly 98% of these stillbirths occur in low- and middle-income countries (LMIC), where the majority could be prevented with known interventions [2, 3]. We have reported on stillbirth rates from a population-based study in LMIC in the Global Network for Women’s and Children’s Health Research and found stillbirth rates ranging from 18 per 1000 births in Kenya to 44 per 1000 births in Pakistan [4, 5]. Another population-based study from sites in south Asia and sub-Saharan Africa, known as the Alliance for Maternal and Newborn Health Improvement (AMANHI), found similar stillbirth rates ranging from 35 per 1000 births in the Asian sites compared to 17 per 1000 births in the African sites [6].

In addition to challenges with complete reporting of stillbirths in resource-limited areas, the lack of reliable estimates of the medical causes of stillbirth, especially in LMIC, has been of concern [7]. While several research efforts are currently underway to determine the causes of stillbirth [8, 9], in practice, most stillbirths remain undocumented and when recorded, few characteristics of the stillbirths are available. As one step to better understand stillbirth, efforts have been made to record the timing of stillbirth [10]. Specifically, whether a stillbirth occurred prior to labor, also known as antepartum stillbirths, or during labor (intrapartum stillbirths) has important implications. Estimates from the Global Network and AMANHI have suggested that more than half of stillbirths may occur in the intrapartum period and are generally considered preventable [4, 5].

The quality of obstetric care is highly correlated with risk of stillbirth. Both the lack of access to antenatal care and the poor quality of care during labor and delivery have been associated with increased risk of stillbirth. In particular, the low population rates of Cesarean section have been correlated with increased stillbirth risk [11]. In high-resource settings with high quality obstetric care and access to Cesarean section, intrapartum stillbirths have largely been eliminated [12].

With an urgent need for data to help document the rates [13], timing and causes of stillbirth in LMIC, we have previously reported on stillbirths in a population-based study conducted in six countries in south Asia, sub-Saharan Africa, and Guatemala [4, 5, 14]. Here we seek to update our reports with estimates of stillbirth rates, timing and causes from 2010 through 2018 in a prospective study from the *Eunice Kennedy Shriver* National Institute of Child Health and Human Development Global Network for Women’s and Children’s Health Research (Global Network) [14, 15].

## Methods

The Global Network’s Maternal Newborn Health Registry (MNHR) is a prospective observational study that includes all pregnant women and their outcomes in defined geographic communities (clusters). For this study, sites in the Democratic Republic of Congo (DRC) (North and South Ubangi Provinces), western Kenya, Zambia (Kafue and Chongwe), Pakistan (Thatta), India (Belagavi and Nagpur) and Guatemala (Chimaltenango) were included. Each site had between 10 and 24 study clusters, which are defined geographic areas with approximately 300–500 annual births [15].

The MNHR staff, generally community health workers or nurses, known as registry administrators (RAs), attempted to identify and screen all pregnant women residing or delivering in the study communities within 48 h of delivery. At enrollment, basic demographic information was recorded, and a follow-up visit conducted within 48 h of the delivery to obtain birth outcomes, as described in detail elsewhere [15]. The study outcome data were based on medical record review, as well as interviews with birth attendants and when applicable, the family. In addition to the prospective enrollment of pregnant women, several measures were taken to ensure accuracy of the stillbirth data, including supervisory oversight of RAs’ data, review of the ratio of stillbirth to early neonatal death to identify any potential biases, and training and review of definitions.

### Study definitions

Stillbirth was defined using a modified World Health Organization (WHO) criteria of fetal deaths occurring at ≥20 weeks gestation (or for those without gestational age available ≥500 g) [16]. Macerated stillbirths were defined as those with visible signs of maceration including skin or soft tissues changes such as skin sloughing or discoloration.

In 2014, the Global Network MNHR study introduced an additional data collection tool to facilitate classification of the cause of stillbirth. Using data from the supplemental form as well as clinical information in the MNHR, a model was used to estimate one primary cause of stillbirth [17, 18]. Briefly, the hierarchal algorithm first evaluates whether the stillbirth was associated with fetal trauma (i.e., accident). Next, the presence of a major (visible) congenital anomaly is assessed for potential causality; ultrasound and other more sophisticated techniques were not routinely used. If neither is present and signs of maternal or fetal infection are observed, the stillbirth is classified as infection. If none of these are present and any maternal or fetal condition associated with intrauterine asphyxia (including preeclampsia/eclampsia, hemorrhage, obstructed or prolonged labor) is present, asphyxia is defined as the cause. Finally, preterm birth is considered the cause of death if none of the prior conditions were present and the stillbirth was less than 32 weeks gestation. If none of the conditions were present, the cause of stillbirth is classified as unknown.

Risk factors for stillbirth were prospectively defined based on literature review of potential factors associated with stillbirth in low-resource settings. These included maternal clinical conditions, antenatal and delivery care as well as characteristics of the fetus that were collected as part of our routine registry.

### Data analyses

A team at each research site supervised local data collection and provided the initial review of the data collected. Then, data were entered at each study site and transmitted through a secure process to the central data coordinating center, RTI International (RTI, Durham, NC). Descriptive analyses were performed as well as log binomial models using general estimation equations to account for the correlation of outcomes within cluster to estimate relative risk of stillbirth. The incidence of stillbirth was calculated as the number of stillbirths per 1000 births (live and stillbirths > 500 g) The models which evaluated stillbirths by year were limited to those clusters which collected data within the MNHR throughout the full study period, as several sites changed the number of clusters during the study period. All data analyses were done with SAS software v.9.4 (Cary, NC).

### Ethics approval

Each research site obtained local approval by the ethics review committees (INCAP, Guatemala; University of Zambia, Biomedical Research Ethics Committee, Zambia; Moi University School of Medicine, Kenya; University of Kinshasa, DRC; Aga Khan University; KLE University’s Jawharal Nehru Medical College, Belagavi, India; Lata Medical Research Foundation, Nagpur, India), the institutional review boards by partner U.S. universities and the data coordinating center (RTI). All pregnant women included in the registry provided informed consent for participation in the study.

## Results

**Fig. 1:**
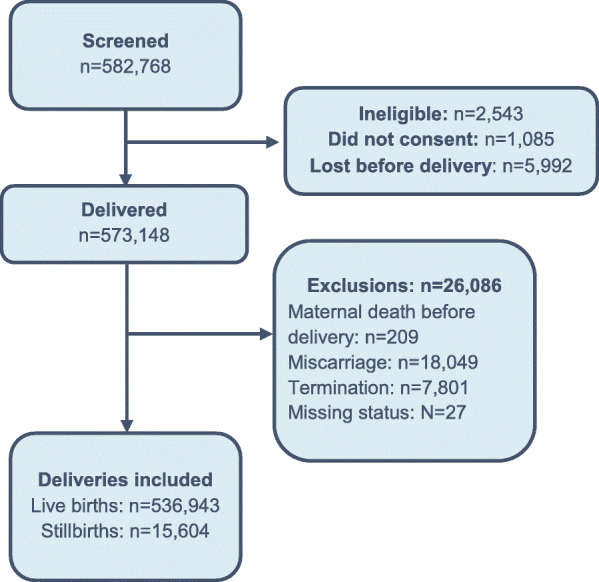
Enrollment diagram for the Maternal and Newborn Health Registry, 2010–2018

From January 2010 through December 2018, a total of 582,768 women were screened, and 573,148 (98.3%) were eligible and consented (Fig. 1). After exclusion of women with a miscarriage, termination of pregnancy or missing delivery outcomes, 552,547 (96.4%) deliveries were included in the analyses. This included 536,943 live births and 15,604 stillbirths, a stillbirth rate of 28.2 per 1000 births during the study period.

Table 1 describes the stillbirth characteristics by study site. The stillbirth rates for the study period ranged from a little over 19 per 1000 births in the Guatemalan and Zambian sites to about 53 per 1000 in the Pakistan site. Of all stillbirths, nearly two-thirds (63.4%) did not have visible signs of maceration, while nearly one-third (29.1%) had signs of maceration. The status of maceration for 7.5% of the stillbirths was unknown.

**Table 1:** Global Network stillbirth rates by site, 2010–2018

	Total Births	Stillbirths	Stillbirth rate/1000 births	Macerated stillbirth, %	Non-macerated stillbirth, %	Maceration status unknown, %
**Total, N**	**552,547**	**15,604**	**28.2**	**29.1%**	**63.4%**	**7.5%**
**African sites**	**170,246**	**4053**	**23.8**	**30.2%**	**67.0%**	**2.9%**
**Asian sites**	**298,830**	**9941**	**33.3**	**30.1%**	**60.1%**	**9.8%**
**Rates by site**						
Guatemala	83,471	1610	19.3	20.4%	74.3%	5.2%
DRC	32,143	1249	38.9	35.1%	63.5%	1.4%
Zambia	63,188	1206	19.1	36.0%	62.4%	1.6%
Kenya	74,915	1598	21.3	21.9%	73.2%	4.9%
Belagavi	123,646	3012	24.4	36.0%	63.5%	0.5%
Nagpur	83,095	2007	24.2	19.2%	68.9%	11.9%
Pakistan	92,089	4922	53.4	30.9%	54.4%	14.7%

Both prematurity and birth weight < 2500 g were significantly associated with risk of stillbirth (Table 2). Multiple gestation, breech presentation and male gender were also statistically significant risk factors for stillbirth.

**Table 2:** Fetal Characteristics by Risk of Stillbirth, Global Network Sites 2010–2018

	Stillbirth	Live birth	RR^1^ for stillbirth vs. live birth (95% CI)
Births, N	15,604	536,943	
GA, N (%)	14,695	521,904	
Preterm	8786 (59.8)	63,785 (12.2)	8.6 (7.8, 9.5)
Term	5909 (40.2)	458,119 (87.8)	1.0
Birth weight, N (%)	14,457	536,819	
< 2500 g	9121 (63.1)	71,369 (13.3)	9.3 (8.4, 10.4)
≥ 2500 g	5336 (36.9)	465,450 (86.7)	1.0
Gender, N (%)	14,037	536,883	
Male	7777 (55.4)	276,529 (51.5)	1.2 (1.1, 1.2)
Female	6260 (44.6)	260,354 (48.5)	1.0
Multiple, N (%)	14,932	536,880	
Yes	865 (5.8)	9779 (1.8)	2.9 (2.7, 3.2)
No	14,067 (94.2)	527,101 (98.2)	1.0
Breech presentation, N (%)	15,535	536,413	
Yes	1259 (8.1)	10,829 (2.0)	3.6 (3.3, 4.1)
No	14,276 (91.9)	525,584 (98.0)	1.0

^1^Relative Risks are modeled assuming a binomial distribution with a log link and accounting for clustering within each MNH site

Most women were 20–35 years of age, with higher risk of stillbirth among those > 35 years of age (Table 3). Additionally, lower educational status was associated with risk of stillbirth. Women who were of parity 1–2 had lower risk of stillbirth relative to nulliparous women or women with greater than 2 prior pregnancies. Finally, those women with a pregnancy loss in the prior pregnancy also had a higher risk for stillbirth (RR 2.4, 95% CI 2.2 2.6). Women who had fewer antenatal care visits and those delivered by family members compared to those with a physician delivery had over twice the risk of stillbirth (Table 4). Also compared to physician deliveries, women delivered by a nurse or other skilled health workers (RR 0.6, 95% CI 0.5, 0.7) or a traditional birth attendant (RR 0.5, 95% CI 0.4, 0.6). had a lower risk of stillbirth. Delivery in clinic compared to hospital and home deliveries was associated with a lower risk of stillbirth (RR 0.5, 95% CI 0.5–0.6). Cesarean section was also associated with lower risk of stillbirth (RR 0.6, 95% CI 0.6–0.7) compared to vaginal delivery. Assisted vaginal delivery was associated with a higher risk of stillbirth (RR 2.0, 95% CI 1.4, 2.9).

**Table 3:** Maternal Characteristics and Risk of Stillbirth in Global Network sites, 2010–2018

	Stillbirth	Live Birth	RR^a^ for stillbirth vs. live birth (95% CI)
Maternal age, N (%)	15,576	535,960	
< 20	1690 (10.9)	68,421 (12.8)	1.0 (1.0, 1.1)
20–35	12,724 (81.7)	443,093 (82.7)	1.0
> 35	1162 (7.5)	24,446 (4.6)	1.6 (1.5, 1.8)
Education, N (%)	15,558	535,272	
No formal education	6099 (39.2)	126,678 (23.7)	1.7 (1.5, 1.9)
Primary	3968 (25.5)	157,969 (29.5)	1.3 (1.2, 1.4)
Secondary	4763 (30.6)	213,538 (39.9)	1.2 (1.1, 1.3)
University+	728 (4.7)	37,087 (6.9)	1.0
Parity, N (%)	15,445	533,588	
0	4972 (32.2)	172,516 (32.3)	1.3 (1.2, 1.3)
1–2	5273 (34.1)	225,205 (42.2)	1.0
> 2	5200 (33.7)	135,867 (25.5)	1.4 (1.3, 1.4)
Last pregnancy resulted in a live birth, N (%)	10,469	361,010	
Yes	9136 (87.3)	341,778 (94.7)	1.0
No	1333 (12.7)	19,232 (5.3)	2.4 (2.2, 2.6)

^a^Relative Risks are modeled assuming a binomial distribution with a log link and accounting for clustering within each MNH site

**Table 4:** Antenatal and Obstetric Care Characteristics and Risk of Stillbirth, Global Network Sites 2010–2018

	Stillbirth	Live Birth	RR^a^ for stillbirth vs. live birth (95% CI)
Births^a^, N	15,604	536,943	
ANC visits, N (%)	11,895	419,017	
0	864 (7.3)	9221 (2.2)	3.5 (2.9, 4.2)
1–2	3919 (32.9)	70,460 (16.8)	2.2 (1.9, 2.6)
≥ 3	7112 (59.8)	339,336 (81.0)	1.0
Birth attendant, N (%)	15,589	536,894	
Physician	6219 (39.9)	192,182 (35.8)	1.0
Nurse/Midwife/HW	4177 (26.8)	200,467 (37.3)	0.6 (0.5, 0.7)
TBA	2860 (18.3)	117,774 (21.9)	0.5 (0.4, 0.6)
Family/Other	2333 (15.0)	26,471 (4.9)	2.1 (1.7, 2.6)
Delivery location, N (%)	15,602	536,873	
Hospital	7438 (47.7)	234,257 (43.6)	1.0
Clinic	3380 (21.7)	167,496 (31.2)	0.5 (0.5, 0.6)
Home/Other	4784 (30.7)	135,120 (25.2)	0.9 (0.8, 1.0)
Delivery mode, N (%)	15,597	536,935	
Vaginal	13,907 (89.2)	457,414 (85.2)	1.0
Vaginal assisted	404 (2.6)	5071 (0.9)	2.0 (1.4, 2.9)
C-section	1286 (8.2)	74,450 (13.9)	0.6 (0.6, 0.7)

^a^Relative Risks are modeled assuming a binomial distribution with a log link and accounting for clustering within each MNH site

Cause of stillbirth classification was available for the 7188 stillbirths enrolled since the classification system was introduced in 2014 (17, 18). Across all sites, birth asphyxia was the most frequent cause of stillbirth, accounting for 44.0% (ranging from 25.0% in the DRC to 57.7% in the Zambia sites) (Table 5). Infectious causes were the second most common cause, accounting for 22.2% of the stillbirths (ranging from 25.3 to 60.2% in the African sites, but substantially lower in the Indian sites (e.g., 2.3% in Nagpur, India). Congenital anomalies were a less frequent cause, except for the Belagavi, India site, in which 22.7% of the stillbirths were attributed to a major anomaly. The majority (51.9%) of non-macerated stillbirths were due to asphyxia whereas a plurality of macerated stillbirths (35.0%) were attributed to infectious causes (Table 6). Finally, we evaluated the frequency of the maternal conditions that were identified with stillbirth by the fetal cause (Table 7). Among women with a stillbirth and obstructed or prolonged labor, the primary cause of stillbirth was attributed to asphyxia. Similarly, asphyxia was deemed the cause for nearly 78% of women with antepartum hemorrhage. Hypertensive disease of pregnancy, breech and maternal infections were also associated with stillbirths attributed to asphyxia.

**Table 5:** Cause of Stillbirth, Global Network Sites 2014–2018

	Africa			Latin America	Asia			Total
	DRC	Zambia	Kenya	Guatemala	Belagavi	Nagpur	Pakistan	
Cause of Stillbirth^a^, N (%)	1246	588	761	914	1021	809	1849	7188
Trauma	0 (0.0)	0 (0.0)	0 (0.0)	0 (0.0)	0 (0.0)	0 (0.0)	1 (0.1)	1 (0.0)
Congenital Anomaly	29 (2.3)	16 (2.7)	20 (2.6)	88 (9.6)	232 (22.7)	59 (7.3)	87 (4.7)	531 (7.4)
Infection	750 (60.2)	149 (25.3)	239 (31.4)	41 (4.5)	66 (6.5)	19 (2.3)	330 (17.8)	1594 (22.2)
Asphyxia	312 (25.0)	339 (57.7)	365 (48.0)	417 (45.6)	468 (45.8)	431 (53.3)	830 (44.9)	3162 (44.0)
Prematurity	55 (4.4)	25 (4.3)	32 (4.2)	79 (8.6)	106 (10.4)	101 (12.5)	136 (7.4)	534 (7.4)
Other/Unknown	100 (8.0)	59 (10.0)	105 (13.8)	289 (31.6)	149 (14.6)	199 (24.6)	465 (25.1)	1366 (19.0)

^a^Cause of stillbirth collected from 2014

**Table 6:** Cause of stillbirth by signs of maceration, 2014–2018

Variables	Signs of Maceration		
	Macerated	Non-Macerated	Maceration Status Unknown
Cause of Stillbirth, N (%)	2194	4940	54
Trauma	0 (0.0)	1 (0.0)	0 (0.0)
Congenital Anomaly	196 (8.9)	332 (6.7)	3 (5.6)
Infection	767 (35.0)	810 (16.4)	17 (31.5)
Asphyxia	582 (26.5)	2565 (51.9)	15 (27.8)
Prematurity	0 (0.0)	534 (10.8)	0 (0.0)
Unknown	649 (29.6)	698 (14.1)	19 (35.2)

**Table 7:** Cause of Stillbirth by Maternal Condition

	Maternal Condition Present				
Variables	Evidence of hypertensive disease/ pre-eclampsia/ eclampsia	Antepartum Hemorrhage	Obstructed or Prolonged Labor	Breech or Transverse Lie	Maternal Infection
Stillbirths, N	696	815	1482	699	760
Cause of Stillbirth, N (%)					
Trauma	1 (0.1)	0 (0.0)	0 (0.0)	0 (0.0)	0 (0.0)
Congenital Anomaly	24 (3.4)	19 (2.3)	44 (3.0)	39 (5.6)	31 (4.1)
Infection	87 (12.5)	161 (19.8)	279 (18.8)	131 (18.7)	623 (82.0)
Asphyxia	551 (79.2)	634 (77.8)	1153 (77.8)	388 (55.5)	100 (13.2)
Prematurity	9 (1.3)	0 (0.0)	2 (0.1)	38 (5.4)	6 (0.8)
Unknown	24 (3.4)	1 (0.1)	4 (0.3)	103 (14.7)	0 (0.0)

**Fig. 2:**
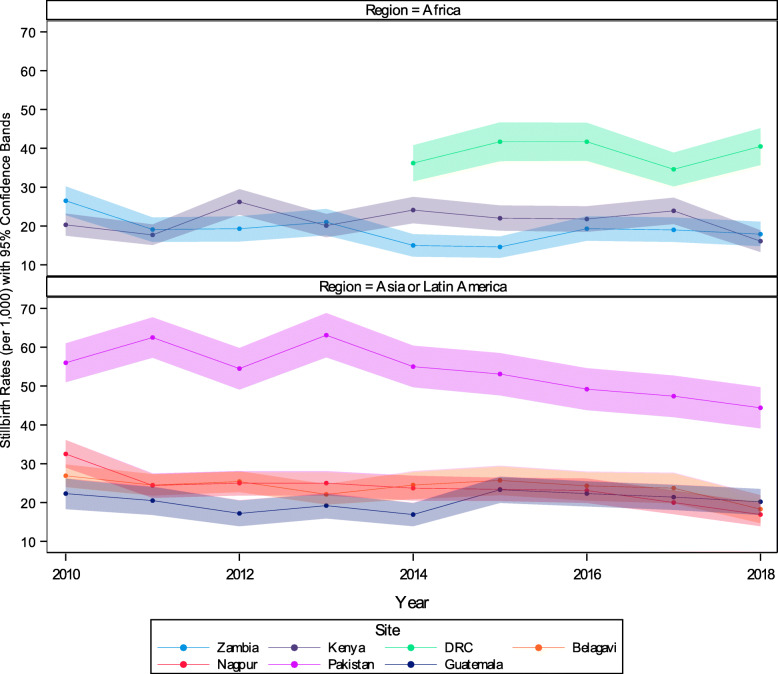
Global Network Stillbirth Rates by Site, 2010–2018

Among study clusters that continued from 2010 to 2018, we visually assessed the stillbirth rates by year (Fig. 2). With the exceptions of the Pakistan site, which had the highest rates of stillbirth and appeared to have a substantial decrease from 2010 to 2018 (from 56.0 per 1000 to 44.4 per 1000) and the Nagpur, India site (which recorded stillbirth rates of 32.5 per 1000 in 2010 compared to 16.9 in 2018), across the other sites, only modest decreases in the stillbirth rates between 2010 and 2018 were reported.

## Discussion

In this population-based study from the Global Network, the overall stillbirth rate was 28.2 per 1000 births, rates that are ten-fold higher than those reported from high-resource settings. The rates of stillbirth varied substantially between the sites, with the Pakistani site documenting the highest stillbirth rate of about 50 per 1000 births followed by the DRC site at about 40 per 1000 births while the other sites ranged between 20 to 25 stillbirths per 1000 births. However, all stillbirth rates were substantially higher than the rates of 2 to 5 per 1000 births currently observed in high-resource settings [12].

Similar to our prior results, a substantial proportion of these stillbirths - about 40% were term and a similar proportion had a birthweight ≥2500 g, representing newborns who should potentially survive even in low-resource settings, if born alive [5]. In the U.S. and other high-income countries, virtually all deliveries with similar characteristics result in live-born infants who survive [12].

Another important finding was that the women most at risk for stillbirth continue to be those with less education and with less antenatal care. Women who had multiple pregnancies and breech presentations in these low-resource settings also experienced substantially increased risk of stillbirth. While these are generally considered risk factors in pregnancy, most can be successfully overcome with access to quality health care services that can identify and appropriately manage these conditions.

The high proportion of stillbirths attributed to congenital anomalies in the Belagavi, India site merits discussion. Prior studies have found a high proportion of consanguinity in the Belagavi, India site which may partially explain the large percentage of stillbirths attributed to congenital anomalies found in that site [19]. However, consanguinity is also relatively common in the other Indian and Pakistan sites, and these sites reported lower percentages of stillbirths caused by congenital anomalies. Further study of the relationship of congenital anomalies to stillbirths in the Asian sites is needed to understand these findings. Additionally, while our data might suggest that being delivered by a nurse or TBA or delivering out of a hospital was associated with lower stillbirth risk, most likely, complicated cases or those with a known stillbirth were more often referred to a physician for a hospital delivery. Further in-depth study will need to be undertaken to confirm a causal relationship.

Finally, as we examined cause of stillbirth using our algorithm [18], the plurality of stillbirths was attributed to birth asphyxia followed by infectious causes. Maternal conditions including hemorrhage, hypertensive disease and infections likely were associated with many of the stillbirths. We have previously emphasized that stillbirths attributed to asphyxia using our algorithm may be attributed to a maternal cause, such as preeclampsia or a placental cause such as fibrosis, necrosis or infarction, in other systems [20, 21]. Assignment of a cause often depends on the clinical training of the coder or the classification system used, rather than underlying differences in cause of death.

Most of the conditions related to stillbirth identified in the Global Network sites are generally either preventable or manageable with quality antenatal and intrapartum care. In contrast, in high-income countries, most stillbirths are of very early gestational ages, are macerated and attributed to conditions such as placental failure or congenital anomalies and the fetuses are often not considered viable given the current treatment options [12].

There were several limitations to the study. First, for determining the cause of stillbirth, only clinical and routine laboratory data were available to inform the cause of death algorithm. Thus, while ideally more comprehensive assessment of stillbirth would include fetal cause, maternal conditions as well as an assessment of the placenta, our results only include the clinically observed fetal and maternal conditions. As a result, infections, which may not have been readily apparent by clinical observation or without placental examination, may have been under-reported, especially in the births that took place at home or in a clinic. Additionally, the study may have missed more subtle anomalies so anomalies may have been under-reported across sites. However, given those limitations, we did utilize an algorithm that defined cause consistently across sites to reduce potential bias. Since many of the births occurred at home or in understaffed facilities, fetal motion, respiration or heart rate in live born infants may not have been observed and the infant misclassified as a stillbirth [22].

Another limitation of the study may have been related to our ability to evaluate the trends in stillbirth rates over time. Over the last decade of the MNHR, improvements have occurred in ascertainment of several measures that would potentially impact detection of stillbirth [23–26]. For example, over the years, the study sites have increasingly identified and enrolled women earlier in their pregnancy, ultrasound scanning for gestational age determination has increased and measurements of birth weight with more accurate scales have increased. Extensive training in newborn resuscitation also occurred and infants previously considered stillborn were now being correctly classified as live births [26].

However, despite these limitations, to our knowledge, the Global Network MNHR remains one of the largest, prospective, population-based pregnancy registries worldwide. Across the sites, a common protocol and methodology were used to document the pregnancy outcome, including stillbirth, as well as the characteristics associated with risk of stillbirth in low-resource settings.

## Conclusions

Stillbirth remains an important and often overlooked component of maternal and child health. As stillbirth rates in LMIC are high and appear to have changed little in most of our sites in recent years, increasing efforts to correctly classify the burden of stillbirth in LMIC are indicated. In addition to knowing the true burden of stillbirth, efforts to better understand the causes of stillbirth are needed to inform interventions to substantially reduce stillbirths globally.

## Data Availability

Study data will be available through the NICHD data and specimen hub (NDASH available at https://dash.nichd.nih.gov/).

## Abbreviations

LMIC: Low- and middle-income countries
AMANHI: Alliance for Maternal and Newborn Health Improvement
MNHR: Maternal Newborn Health Registry
DRC: Democratic Republic of Congo
RAs: Registry administrators
WHO: World Health Organization
